# Genotoxic and Carcinogenic Potential of Compounds Associated with Electronic Cigarettes: A Systematic Review

**DOI:** 10.1155/2019/1386710

**Published:** 2019-12-19

**Authors:** Isaac Armendáriz-Castillo, Santiago Guerrero, Antonella Vera-Guapi, Tiffany Cevallos-Vilatuña, Jennyfer M. García-Cárdenas, Patricia Guevara-Ramírez, Andrés López-Cortés, Andy Pérez-Villa, Verónica Yumiceba, Ana K. Zambrano, Paola E. Leone, César Paz-y-Miño

**Affiliations:** Centro de Investigación Genética y Genómica, Facultad de Ciencias de la Salud Eugenio Espejo, Universidad UTE, Quito, 170147, Ecuador

## Abstract

**Background:**

Many studies, comparing the health associated risks of electronic cigarettes with conventional cigarettes focus mainly on the common chemical compounds found between them.

**Aim:**

Review chemical compounds found exclusively in electronic cigarettes and describe their toxic effects, focusing on electronic-cigarette-only and dual electronic-cigarette and conventional cigarette users.

**Data Sources:**

Literature search was carried out using PubMed.

**Study Eligibility Criteria:**

Articles related exclusively to conventional and electronic cigarettes' chemical composition. Articles which reported to be financed from tobacco or electronic cigarettes industries, not reporting source of funding, not related to the chemical composition of electronic and conventional cigarettes and not relevant to tobacco research were excluded.

**Methods and Results:**

Chemical compounds reported in the selected studies were tabulated using the Chemical Abstracts Service registry number for chemical substances information. A total of 50 chemical compounds were exclusively reported to be present in electronic cigarettes. Crucial health risks identified were: eye, skin, and respiratory tract irritation, with almost 50% of incidence, an increment of 10% in cytotoxic effects, when compared to compounds in common with conventional cigarettes and around 11% of compounds with unknown effects to human health.

**Limitations:**

Articles reporting conflicts of interest.

**Conclusions and Implications of Key Findings:**

Despite being considered as less harmful for human health, compounds found in electronic cigarettes are still a matter of research and their effects on health are yet unknown. The use of these devices is not recommended for first time users and it is considered hazardous for dual users.

## 1. Introduction

Electronic cigarettes (e-cigarettes) have been commercially available for more than a decade [1]. They basically consist of a battery-dependent atomizer which heats fluids with or without nicotine to water vapor [1]. According to the US government, the number of high school students that use e-cigarettes increased at 80% in the last year, in consequence, the American Lung Association, which uses its own federal grading system (0–20 points), gave an “F” grade (under 12 points) in the Regulation of Tobacco Products category to the FDA [2, 3].

Many studies worldwide have analyzed health risks associated with chemical compounds found in both e-cigarettes and conventional cigarettes (CC) [4]. However, 34% of these studies stated conflicts of interest, mainly related to being funded by the manufacturers of e-cigarettes or CC [5]. Despite the increase in electronic-cigarette-only users, no study has analyzed health risks associated with compounds found exclusively in e-cigarettes.

Research on genotoxic and carcinogenic effects related to e-cigarettes has been mainly focused on fluid composition and metal heating [5]. Thus, e-liquids are mainly composed of glycols, nicotine, particles, metals, tobacco-specific nitrosamines (TSNAs), carbonyls, volatile organic compounds (VOCs), hydrocarbons, polycyclic aromatic hydrocarbons (PAHs), and phenols [5]. To date, only few nonconflicted studies have associated e-cigarette fluids and vapor composition with the following health risks: genotoxic and cytotoxic to human cells [6, 7], carcinogenic [8], cardiovascular [9] and pulmonary effects [10].

As reported by Pisinger & Døssing, 2014 [1], most studies used CC as reference to study the effects of e-cigarettes on human health. However, health risks, like carcinogenic effects, associated only with e-cigarettes remain unclear and more evidence is needed [2].

Therefore, we performed a comprehensive analysis using select nonconflicted articles to detect chemical compounds only found in e-cigarettes, with the aim to report toxic effects which can lead to different health risks associated with these compounds.

## 2. Methods

### 2.1. Literature Search

**Figure 1:**
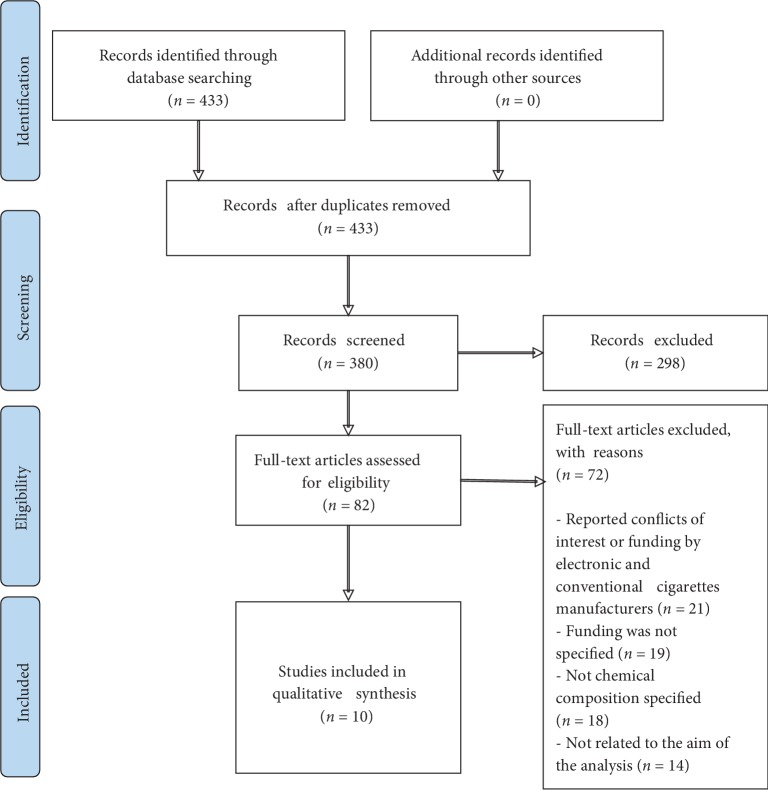
PRISMA flow diagram showing the filtering process of the articles selected to analyze chemical composition of electronic and conventional cigarettes.

In order to screen for hazardous CC and e-cigarettes components, we carried out a literature search using PubMed (https://www.ncbi.nlm.nih.gov/pubmed/) (Supplementary [Supplementary-material supplementary-material-1]). Keyword used for searching articles were: “e-cigarettes chemical composition” and “cigarettes chemical composition”. All authors participated in the literature search, papers selected were discussed and all agreed to consider articles including reviews and research papers with exception of articles where the authors reported to have worked or received funding from tobacco industry or e-cigarette manufacturers, additionally, articles which did not specify sources of funding, articles where chemical composition was not clearly detailed or not related to the aim of this review were also excluded. Risk of bias was assessed in the corresponding sections of the main article, in order to identify conflicts of interest or problems with funding. The number of articles selected and excluded can be observed in the PRISMA flow diagram (Figure 1) [11].

To obtain a comprehensive list of CC chemical compounds with known health effects, we merged 82 compounds from [12], 98 compounds from [13], 50 compounds from [14], 30 compounds from [15], 95 compounds from [16], and 94 compounds from [17]. As a result, a list of 150 chemical compounds was generated. Similarly, a list of 84 compounds only found in e-cigarettes was generated using 29 compounds from [18], 32 compounds from [19], 13 compounds from [20], and 61 compounds from [21].

### 2.2. Nomenclature and Classification

To optimally compare CC and e-cigarettes' chemical compounds, we used the numerical identifier assigned by the Chemical Abstracts Service (CAS) [22]. Compounds without CAS registries were designed as unknown. All compounds were classified according to their carcinogenic potential [23]: group 1 as carcinogenic to humans, group 2A as probably carcinogenic to humans, group 2B as possibly carcinogenic to humans, group 3 as not classifiable as to its carcinogenicity to humans, and group 4 as probably not carcinogenic to humans and “i.e” for compounds with inadequate evidence. Additionally, compounds were classified according to their health associated risk: eyes, skin and respiratory track irritation, mild effects, cardiovascular system problems, carcinogenic, neurotoxic, harmful for animal models, cytotoxic, reproduction or developmental effects, systemic organ irritation and unknown effects for human health.

## 3. Results and Discussion

### 3.1. Identification of e-Cigarettes' Chemical Compounds

**Figure 2:**
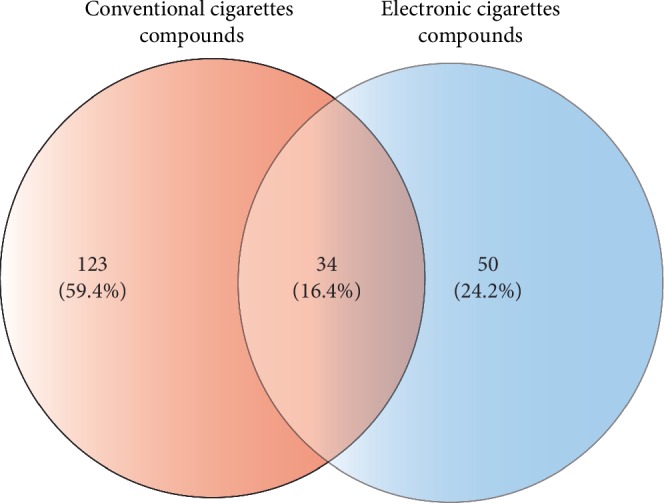
Venn diagram showing the number of common and unique chemical compounds between electronic and conventional cigarette.

To identify chemical compounds exclusively present in e-cigarettes, we first performed a literature review to determine CC and e-cigarettes' chemical compounds having a known impact on human health, articles which reported conflicts of interest or funded by electronic and conventional cigarette manufacturers in the corresponding sections of the main article were excluded. As a result, 234 chemical compounds were found: 150 for CC and 84 for e-cigarettes. When comparing both lists (see Figure 2), we found 34 compounds in common with CC (Supplementary Table 1) and 50 exclusively present in e-cigarettes (Table 1).

### 3.2. Health Associated Risks of Chemical Compounds Found in e-Cigarettes

**Figure 3:**
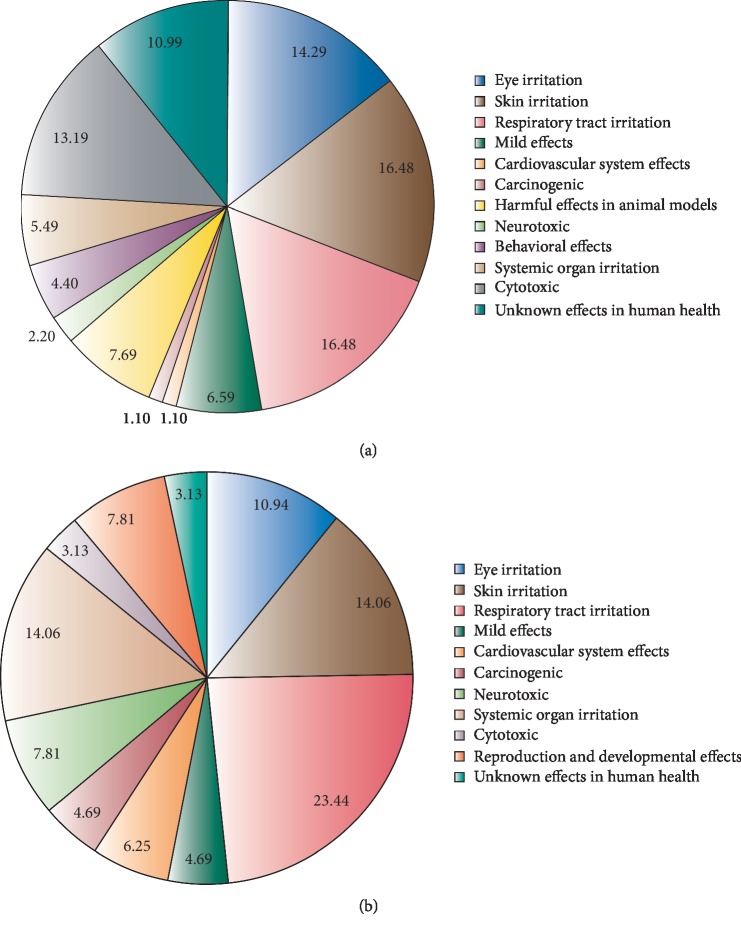
(a) Health risks associated to chemical compounds found exclusively in electronic cigarettes. (b) Health risks associated to chemical compounds from conventional cigarettes.

Figure 3 shows the percentage of health associated risks of chemical compounds present only in e-cigarettes (*n* = 50) and common compounds with CC (*n* = 34). From this analysis, three health risks are the most prevailing between both groups: eye, skin, and respiratory tract irritation, with almost 50% of incidence, while cardiovascular, carcinogenic, and neurotoxic effects are also reported in e-cigarettes' exclusive compounds, which are common health effects of CC smoking according to the Centers for Disease Control and Prevention [42]. There are around 11% of compounds effects of which in human health remain unknown, and around 7.7% have been tested in animal models and proved to be harmful. Finally, cytotoxic effects of e-cigarette compounds (13%) are higher than those present in CC (3%).

From the 50 unique e-cigarette compounds, the effect of around 11% remains unknown for human health (see Figure 3(a)). Most of these are mainly found in e-liquids used to give flavour to the e-cigarettes; for instance, ethyl vanillin is found in the top three products of e-liquids [43]. Several studies have reported that presence of vanillin and cinnamaldehyde in e-liquids is highly related to toxicity [44].

The majority of chemical effects with unknown health effects are present in e-liquids, normally they are safe when digested, but little is known about inhalation of these products [45]. Most e-liquid manufacturers do not include its composition or chemical concentrations in labels, despite knowing that some of these chemicals are proved to be cytotoxic in cellular and animal models [46].

Using MTT (3-(4,5-dimethylthiazol2-yl)−2,5-diphenyltetrazolium bromide) assay, different authors have reported high levels of cytotoxicity of the main compound of e-liquids [47, 48]. A recent study found that vaping effects cause an inflammatory response in lung cells, similar to the response of conventional tobacco smokers and patients with obstructive pulmonary disease [49].

We found concordance between our findings and other studies. For example, P. Callahan-Lyon (2014) reported that the main components of e-liquids, such as glycol and glycerol, when vaporized, can cause throat, mucous membranes, and eye irritation [50]. In addition, Czoli et al. (2019) found similar results when analyzing health associated risks of e-cigarettes in Canadian populations [51].

Despite being reported as safer than CC, e-cigarette compounds are known to induce toxicological effects in human health that can led to genetic alterations that further initiate cancer progression in animal models [52]. Additionally, well-known carcinogens such as safrole and N´-Nitrosonornicotine have been identified in e-liquids and saliva of e-cigarette users, respectively [53, 54].

### 3.3. Disadvantages of the Use of e-Cigarettes

Behavioral effects related to nicotine addiction are regularly seen in first time vapers; for example, different studies found traces of nicotine in e-liquids labeled as free-nicotine [55]. This can lead first-time users of e-cigarettes, normally teenagers, to the need of increasing nicotine concentration in e-liquids and progression to CC [56].

Over the past years, the use of e-cigarettes is on the rise, literature confirms that their use is intended as a transitional stage for quitting smoking [57]. However, because e-cigarettes are a technological novelty and have a high publicity behind them, their use has been reported in many first-time users which never smoked, whom when surveyed, do not know about any associated health effect causes by its chemical compounds [57].

A matter of concern of e-cigarette effects occurs on dual users. These users are reported to generate more addiction to nicotine than e-cigarette only users, however, for the last group, the level of nicotine absorbed is higher, because they vape more often than regular smokers [58]. Accordingly, a study on biomarkers of exposure to toxics, such as carbon monoxide (CO), 1-hydroxypyrene (1-HOP), and 4-(methylnitrosamino)-1-(3-pyridyl)-1-butanol (NNAL), showed that dual-users presented higher values of these biomarkers when compared to e-cigarette only users [59].

## 4. Conclusions

Despite the fact that e-cigarettes exclusive compounds showed less incidence of health related risks when compared to CCs, it is not enough to conclude that its use is safer. There are many cytotoxic and genotoxic effects still unknown related to different compounds of e-cigarettes, especially the ones included in e-liquids, which can be potentially toxic and carcinogenic to humans. Different studies showed how the use of e-liquids can lead to an increasing nicotine addiction and a possible progression to conventional tobacco in first time e-cigarette users. Furthermore, dual users are a group of high risk, not only because of higher nicotine absorption, but, because the health related effects found in common compounds between e-cigarettes and conventional cigarettes will be increased. Finally, due to the lack of experimental evidence regarding health effects associated to e-cigarettes, the use of these devices is not recommended to first time users.

## Figures and Tables

**Table 1:** Chemical compounds exclusively reported in electronic cigarettes liquids.

Name	Cas registry number	Health effect	Classification	Reference
(+)-aromadendrene	489-39-4	Cytotoxic/skin irritation	i.e	[24]
				[25]
(Z)-3-Hexen-1-ol	928-96-1	Eye irritation	i.e	[24]
1-Methyl phenanthrene	832-69-9	Cytotoxic/Eye irritation/Skin irritation	Group 3	[24]
				[26]
1,3-Butanediol	107-88-0	Low concern based on experimental and modeled data	i.e	[27]
1,3-Propanediol	504-63-2	Not a significant hazard via inhalation of either the gas phase or a gas/aerosol mixture	i.e	[27]
				[28]
2-Acetylpyrrole	1072-83-9	Skin irritation	i.e	[24]
2,3-Dimethylpyrazine	5910-89-4	Cytotoxic	i.e	[24]
2,3-Pentanedione	600-14-6	Skin irritation/ eye irritation/ systemic organ irritation	i.e	[24]
2,3,5-Trimethylpyrazine	14667-55-1	Cytotoxic	i.e	[24]
3-Methyl-1-butanol	123-51-3	Cytotoxic/ skin irritation/ eye irritation/ respiratory tract irritation	i.e	[24]
				[29]
Acetic acid	64-19-7	Respiratory tract irritation	i.e	[26]
Benzyl acetate	140-11-4	Cytotoxic/ eye irritation/ respiratory tract irritation	Group 3	[29]
Benzyl alcohol	100-51-6	Cytotoxic	i.e	[24]
Butyl butyrate	109-21-7	Eye irritation/mild effects/ behavioral Effects	i.e	[29]
Camphor	76-22-2	Cytotoxic/ neurotoxic/ systemic organ irritation/ mild effects/ behavioral effects	i.e	[24]
Cinnamaldehyde	104-55-2	Eye irritation/ respiratory tract irritation/ systemic organ irritation	i.e	[29]
Cinnamyl alcohol	104-54-1	Unknown effects in human health	i.e	[24]
Coumarin	91-64-5	Behavioral effects/ systemic organ irritation	Group 3	[29]
Methyl cyclopentenolone	80-71-7	Unknown effects in human health	i.e	[24]
Diacetyl	431-03-8	Eye irritation/ skin irritation	i.e	[29]
Diethylene glycol	111-46-6	Systemic organ irritation/ skin irritation	i.e	[29]
Ethyl butyrate	105-54-4	Mild effects/ behavioral effects	i.e	[29]
				[27]
Ethyl maltol	4940-11-8	Cytotoxic	Unknown	[29]
Ethyl vanillin	121-32-4	Unknown effects in human health	Unknown	[29]
Ethylene glycol	107-21-1	Harmful effects in animal models	Unknown	[30, 31]
Glycerin	56-81-5	Eye irritation/ skin irritation/ respiratory tract irritation	Unknown	[1, 32]
Hydroxyacetone	116-09-6	Cytotoxic	i.e	[33]
i-Butyric acid	79-31-2	Respiratory tract irritation	i.e	[29]
Isobutyl acetate	110-19-0	Eye irritation/ skin irritation / respiratory tract irritation/ mild effects	i.e	[29]
Isoamyl acetate	123-92-2	Eye irritation/ skin irritation / respiratory tract irritation	Unknown	[26]
Isopentyl isovalerate	659-70-1	Harmful effects in animal models	i.e	[25]
L-Menthyl acetate	89-48-5	Respiratory tract irritation	i.e	[26]
Limonene	138-86-3	No evidence of carcinogenic activity in rats or human	i.e	[34]
Maltol	118-71-8	Cytotoxic	Unknown	[26]
Menthone	89-80-5	Harmful effects in animal models	i.e	[29]
Methyl anthranilate	134-20-3	Unknown effects in human health	i.e	[29]
				[25]
Methyl cinnamate	103-26-4	Unknown effects in human health	i.e	[35]
Methyl salicylate	119-36-8	Neurotoxin / cardiovascular effects	Unknown	[25]
Myosmine	532-12-7	Carcinogenic	Unknown	[36]
n-Hexanol	111-27-3	Harmful effects in animal models	i.e	[29]
Nicotyrine	487-19-4	Unknown effects in human health	i.e	[37]
o-Tolualdehyde	529-20-4	Harmful effects in animal models/ unknown effects in human health	Unknown	[26]
				[37]
p-Cymene	99-87-6	Skin irritation/ mild effects	i.e	[26]
Propylene Glycol	57-55-6	Respiratory tract irritation	Unknown	[38]
Safrole	94-59-7	Harmful effects in animal models	Group 2B	[26]
Thujone (sum of *α*- and *β*-diastereomers)	76231-76-0	Harmful effects in animal models	i.e	[39]
Trans-2-hexen-1-ol	928-95-0	Unknown effects in human health	i.e	Sigma-aldrich safety data sheet
Vanillin	121-33-5	Cytotoxic	Unknown	[40]
*β*–Damascone	23726-93-4	Skin irritation	Unknown	[41]
*γ*–Decalactone	706-14-9	Respiratory tract irritation	i.e	[29]

## Data Availability

All relevant data are fully available within the manuscript and its supplementary materials.

## Supplementary Materials

Supplementary Materials Supplementary Table 1: common chemical compounds found between electronic and conventional cigarettes. Supplementary Material 1: search strategy used for PubMed articles selection. Supplementary Material 2: PRISMA checklist for systematic reviews.
