# Supplementary material for: Genotoxic and Carcinogenic Potential of Compounds Associated with Electronic Cigarettes: A Systematic Review
Source: Biomed Res Int. 2019 Dec 19;2019:1386710. doi: 10.1155/2019/1386710 (PMC6948324; doi:10.1155/2019/1386710)
Supplement: Supplementary Materials — Supplementary Table 1: common chemical compounds found between electronic and conventional cigarettes. Supplementary Material 1: search strategy used for PubMed articles selection. Supplementary Material 2: PRISMA checklist for systematic reviews. [file 1386710.f1.pdf]

Supplementary Table 1: Common chemical compounds found between electronic and conventional cigarettes

NAME	CAS REGISTRY NUMBER	HEALTH EFFECT	CLASSIFICATION	REFERENCE
4-(N-nitroso methylamino)-1-(3-pyridyl)-1-butanone	64091-91-4	Cytotoxic/ Skin irritation/ Respiratory tract irritation/ Mild effects	i.e	[42] [43]
Acetaldehyde	75-07-0	Systemic organ irritation	Group 2B	[44]
Acetone	67-64-1	Respiratory tract irritation/ Eye irritation/ Skin irritation/ Systemic organ irritation	Group 3	[44] [43]
Acrolein	107-02-8	Respiratory tract irritation/ Eye irritation/ Systemic organ irritation	Group 3	[44] [45]
Acetoin	513-86-0	Cytotoxic/ Skin irritation/ Eye irritation	i.e	[42]
Anabasine	494-52-0	Respiratory tract irritation/ Mild effects/ Skin irritation	i.e	[42]
Anatabine	581-49-7	Unknown effects in human health	i.e	[42]
Anthracene	120-12-7	Respiratory tract irritation/ Eye irritation	Group 3	[43]
Arsenic	7440-38-2	Skin irritation/ Systemic organ irritation	Group 1	[43, 45]
Benzaldehyde	100-52-7	Skin irritation/ Eye irritation/Respiratory tract irritation	i.e	[43]
Benzene	71-43-2	Cytotoxic/ Cardiovascular effects	Group 1	[45] [46] [47]
Butyraldehyde	123-72-8	Respiratory tract irritation/ Skin irritation/ Mild effects	i.e	[43]
Cadmium	7440-43-9	Respiratory tract irritation/ Carcinogenic	Group 1	[45] [44, 47]
Chromium	7440-47-3	Respiratory tract irritation/ Skin irritation/ Eye irritation /Reproduction and developmental effects	Group 3	[44, 47]
Cotinine	486-56-6	Eye irritation/ Skin irritation/ Respiratory tract irritation	i.e	[42]

Crotonaldehyde	123-73-9	Eye irritation/ Respiratory tract irritation/ Systemic organ irritation	Group 3	[45]
Ethylbenzene	100-41-4	Systemic organ irritation/ Eye irritation/ Respiratory tract irritation/ Skin irritation	Group 2B	[46]
Formaldehyde	50-00-0	Respiratory tract irritation/ Eye irritation/ Skin irritation/ Mild effects	Group 1	[46] [43]
Lead	7439-92-1	Neurotoxin / Cardiovascular effects	Group 2	[48]
Manganese	7439-96-5	Neurotoxin	Group 3	[46]
Menthol	89-78-1	Skin irritation/ Respiratory tract irritation	Unknown	[29]
Methyl ethyl ketone	78-93-3	Eye irritation/ Respiratory tract irritation/ Reproduction and developmental effects	i.e	[46]
Nicotine	54 -11-5	Cardiovascular effects	i.e	[49]
N-nitrosoanabatine (NAT)	71267-22-6	Unknown effects in human health	Group 3	[47]
N-nitrosoanabasine (NAB)	1133-64-8	Unknown effects in human health	Group 3	[47]
N-nitrosonornicotine (NNN)	16543-55-8	Carcinogenic	Group 1	[50]
Nickel	7440-02-0	Respiratory tract irritation / Carcinogenic	Group 1	[17]
Nornicotine	5746-86-1	Skin irritation/ Eye irritation/ Respiratory tract irritation	i.e	[43]
m-Xylene	108-38-3	Respiratory tract irritation/ Neurotoxin / Reproduction and developmental effects	Group 3	[46]
p-xylene	106-42-3	Respiratory tract irritation /Neurotoxin / Reproduction and developmental effects	Group 3	[46]
Pyrene	129-00-0	Skin irritation	Group 3	[43]
Propionaldehyde	123-38-6	Respiratory tract irritation/ Systemic organ irritation/ Cardiovascular effects	i.e	[46]
Phenanthrene	85- 01-8	Systemic organ irritation	Group 3	[51]

Toluene	108-88-3	Neurotoxin/ Reproduction and developmental effects/ Systemic organ irritation	Group 3	[52][53]

Supplementary Material 1: Search strategy used for PubMed articles selection

1. Access to PubMed website: https://www.ncbi.nlm.nih.gov/pubmed/

2. On the search field type: “electronic cigarettes chemical compounds”.

3. On the search field type: “cigarettes chemical compounds”.

4. Filter for papers which titles are related to electronic and conventional cigarettes chemical composition.

5. A total of 433 papers were found, from which, 380 articles were considered for screening.

6. Eligibility criteria included all papers (reviews and research articles) which give relevant information about the chemical composition of electronic and conventional cigarettes, a total of 82 papers were selected after this step.

7. Finally, after applying all the exclusion criteria detailed in the Methods section (funding, conflicts of interest, aim of the systematic review), only 10 papers were considered to be included as relevant literature for chemical composition of electronic and conventional cigarettes.

Supplementary material 2: PRISMA checklist for systematic reviews

Section/topic	#	Checklist item	Reported on page #
**TITLE**			
Title	1	Identify the report as a systematic review, meta-analysis, or both.	Title page
**ABSTRACT**			
Structured summary	2	Provide a structured summary including, as applicable: background; objectives; data sources; study eligibility criteria, participants, and interventions; study appraisal and synthesis methods; results; limitations; conclusions and implications of key findings; systematic review registration number.	1
**INTRODUCTION**			
Rationale	3	Describe the rationale for the review in the context of what is already known.	2
Objectives	4	Provide an explicit statement of questions being addressed with reference to participants, interventions, comparisons, outcomes, and study design (PICOS).	3
**METHODS**			
Protocol and registration	5	Indicate if a review protocol exists, if and where it can be accessed (e.g., Web address), and, if available, provide registration information including registration number.	Supplementary Material page 2
Eligibility criteria	6	Specify study characteristics (e.g., PICOS, length of follow-up) and report characteristics (e.g., years considered, language, publication status) used as criteria for eligibility, giving rationale.	3
Information sources	7	Describe all information sources (e.g., databases with dates of coverage, contact with study authors to identify additional studies) in the search and date last searched.	3
Search	8	Present full electronic search strategy for at least one database, including any limits used, such that it could be repeated.	Supplementary Material page 2
Study selection	9	State the process for selecting studies (i.e., screening, eligibility, included in systematic review, and, if applicable, included in the meta-analysis).	3

Data collection process	10	Describe method of data extraction from reports (e.g., piloted forms, independently, in duplicate) and any processes for obtaining and confirming data from investigators.	3, 4
Data items	11	List and define all variables for which data were sought (e.g., PICOS, funding sources) and any assumptions and simplifications made.	3, 4
Risk of bias in individual studies	12	Describe methods used for assessing risk of bias of individual studies (including specification of whether this was done at the study or outcome level), and how this information is to be used in any data synthesis.	3, 4
Summary measures	13	State the principal summary measures (e.g., risk ratio, difference in means).	
Synthesis of results	14	Describe the methods of handling data and combining results of studies, if done, including measures of consistency (e.g., I^2) for each meta-analysis.	

**Section/topic**	**#**	**Checklist item**	**Reported on page #**
Risk of bias across studies	15	Specify any assessment of risk of bias that may affect the cumulative evidence (e.g., publication bias, selective reporting within studies).	3
Additional analyses	16	Describe methods of additional analyses (e.g., sensitivity or subgroup analyses, meta-regression), if done, indicating which were pre-specified.	
**RESULTS**			
Study selection	17	Give numbers of studies screened, assessed for eligibility, and included in the review, with reasons for exclusions at each stage, ideally with a flow diagram.	4
Study characteristics	18	For each study, present characteristics for which data were extracted (e.g., study size, PICOS, follow-up period) and provide the citations.	4-5
Risk of bias within studies	19	Present data on risk of bias of each study and, if available, any outcome level assessment (see item 12).	
Results of individual studies	20	For all outcomes considered (benefits or harms), present, for each study: (a) simple summary data for each intervention group (b) effect estimates and confidence intervals, ideally with a forest plot.	5-11
Synthesis of results	21	Present results of each meta-analysis done, including confidence intervals and measures of consistency.	
Risk of bias across studies	22	Present results of any assessment of risk of bias across studies (see Item 15).	
Additional analysis	23	Give results of additional analyses, if done (e.g., sensitivity or subgroup analyses, meta-regression [see Item 16]).	5-11
**DISCUSSION**			

Summary of evidence	24	Summarize the main findings including the strength of evidence for each main outcome; consider their relevance to key groups (e.g., healthcare providers, users, and policy makers).	5-11
Limitations	25	Discuss limitations at study and outcome level (e.g., risk of bias), and at review-level (e.g., incomplete retrieval of identified research, reporting bias).	5-11
Conclusions	26	Provide a general interpretation of the results in the context of other evidence, and implications for future research.	12
**FUNDING**			
Funding	27	Describe sources of funding for the systematic review and other support (e.g., supply of data); role of funders for the systematic review.	12

From: Moher D, Liberati A, Tetzlaff J, Altman DG, The PRISMA Group (2009). Preferred Reporting Items for Systematic Reviews and Meta-Analyses: The PRISMA Statement. PLoS Med 6(7): e1000097. doi:10.1371/journal.pmed1000097

For more information, visit: **www.prisma-statement.org**.
